# Digital platforms for ethics review: a mini review

**DOI:** 10.3389/fdgth.2026.1854560

**Published:** 2026-06-09

**Authors:** Brenda Adhiambo Odero, Candice Groenewald

**Affiliations:** 1 School of Law, University of KwaZulu-Natal, Durban, South Africa; 2 Human and Social Capabilities Division, Human Sciences Research Council, Pretoria, South Africa; 3 Research Associate, Psychology Department, University of Western Cape, South Africa

**Keywords:** digital autonomy, digital platforms, digital platforms for ethics review, DPER, ethics review process, RECS, research ethics committees

## Abstract

This mini review examines the emerging use of digital platforms for ethics review (DPER) and the extent to which research ethics committees (RECs) are prepared for digital transformation and operational autonomy. Drawing on a structured analysis of peer-reviewed studies published between 2013 and 2024, the review synthesises the existing research on DPER and examines reported challenges, successes, and implementation recommendations associated with their adoption. In this context, digital autonomy refers to RECs’ readiness to conduct ethics review processes through dedicated digital platforms, supported by adequate infrastructure, stakeholder engagement, institutional capacity, and appropriate data protection and security measures. Findings indicate that digitalisation efforts primarily focus on improving administrative efficiency, protocol tracking, and communication workflows, with reported improvements in processing times and documentation management. However, the literature reveals limited attention to broader system-level governance considerations, including regulatory alignment, infrastructure readiness, data security, and institutional capacity. Across the included studies, there is minimal critical engagement with how DPER reshape accountability structures, transparency mechanisms, and decision-making authority within RECs. The review highlights emerging areas requiring further investigation. This includes governance and regulatory preparedness, variability in institutional digital capacity, and the implications of increasing automation in ethics review processes. Overall, while DPER show promise for strengthening research oversight, the evidence base remains limited in scope and depth. Further empirical and conceptual research is needed to assess institutional and national readiness and to ensure that digital transformation supports, rather than undermines, ethical governance in RECs.

## Introduction

Research ethics review is a basis of responsible research governance, intended to safeguard participants, uphold ethical standards, and ensure institutional accountability (1–3). However, ethics review systems globally face increasing pressures associated with rising research volumes, administrative burden, fragmented communication, and delays in protocol processing. Challenges such as incomplete submissions, inefficient correspondence systems, and prolonged review timelines have been documented across diverse research settings, often affecting research continuity and funding stability (4, 5). Studies examining the implementation of digital platforms for ethics review (DPER) in African contexts report that stakeholders view such systems as enhancing efficiency and communication, while also identifying challenges related to data security, platform design, and training needs (2).

In response to these systemic pressures, DPER have emerged as a proposed mechanism to modernise review workflows by digitising submissions, centralising documentation, and streamlining communication among researchers, secretariats, and review committees. Early reports suggest associated improvements in processing times and record management (6, 7). As such, DPER are often framed as technical solutions to longstanding procedural inefficiencies.

In addition, digitisation in ethics review raises questions that extend beyond workflow optimisation. In this review, digital autonomy refers to the readiness of research ethics committees (RECs) to conduct ethics reviews via dedicated digital platforms, with the necessary infrastructure capacity, stakeholder involvement, institutional support, and adequate privacy and security measures. While often framed as a technical shift, these factors collectively represent a broader governance transition in how ethics oversight is organised and sustained. The adoption of digital platforms introduces issues related to infrastructure readiness, data governance, institutional capacity, and regulatory alignment. These concerns are particularly significant in contexts where RECs operate within resource-constrained institutional environments, including many African research settings where disparities in technological capacity may influence adoption and sustainability (5).

Despite growing interest in digitisation, few studies critically interrogate how digital platforms reshape the governance architecture of ethics review. Most focus on workflow improvements rather than examining how digital systems redistribute authority, alter accountability pathways, or intersect with regulatory frameworks. As such, digital transformation in ethics review is frequently framed as procedural modernisation rather than a governance transition.

This mini-review, therefore, synthesises peer-reviewed literature on digital platforms in ethics review to examine how they are conceptualised, what successes and challenges are reported, and what current evidence suggests regarding readiness for digital autonomy in research ethics governance.

## Review approach

**Figure 1:**
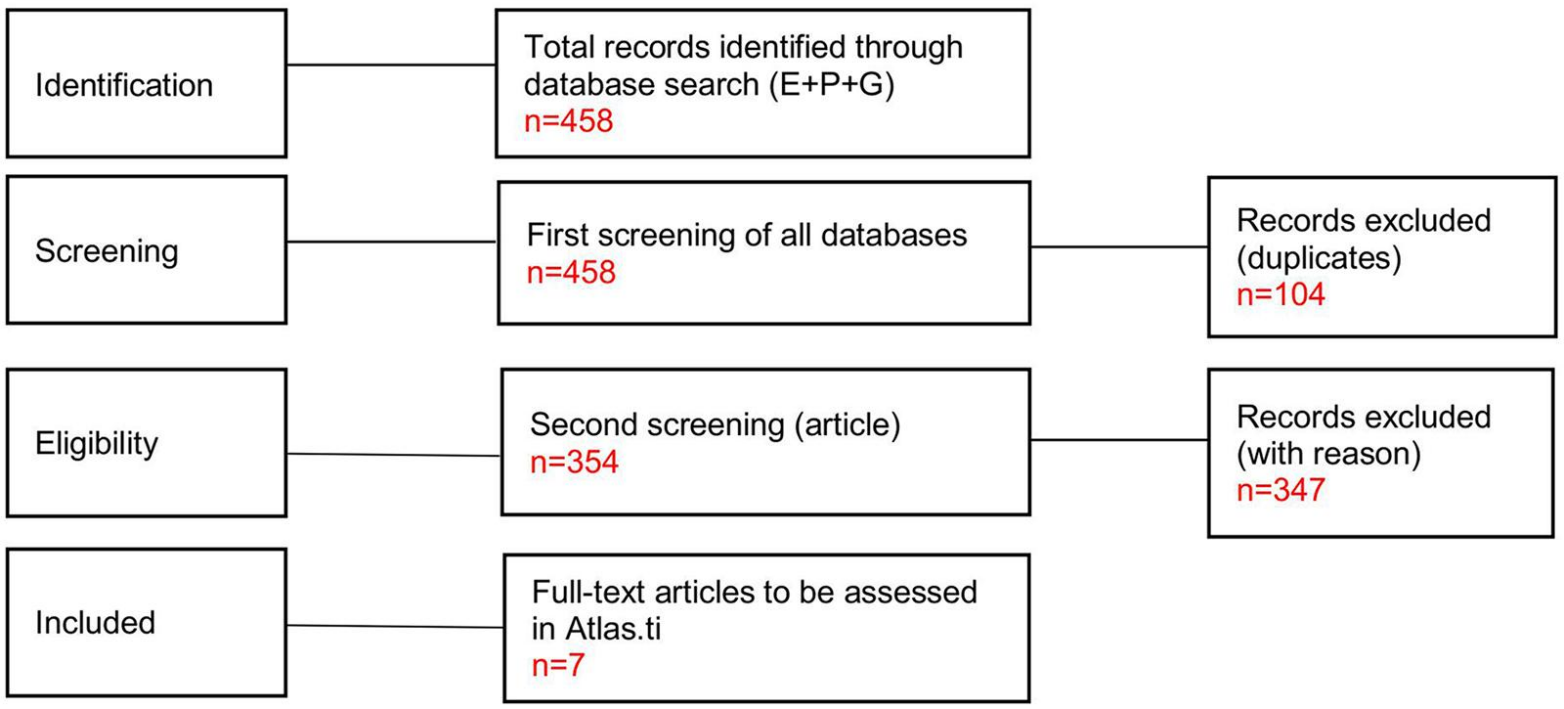
PRISMA diagram to screen articles.

Adhering to PRISMA guidelines (Figure 1), this mini-review draws on a structured search of peer-reviewed literature examining the use of digital platforms in research ethics review processes. Searches were conducted across three major databases: EBSCOHost, ProQuest, and Google Scholar. This was done to capture interdisciplinary scholarship on digital systems used in ethics oversight. The search covered studies published between January 2013 and December 2024.

Search terms reflected variations in digital and online ethics review systems and included the phrases: *online ethics review*, OR *ethics review platform,* OR *electronic ethics review,* OR *automated review platform,* OR *ethics review software,* OR *ethics review system*. Studies were eligible for inclusion if they focused on the use of digital or online platforms specifically within research ethics review processes. Articles were limited to peer-reviewed publications available in English.

The review followed a two-stage screening process. At the identification stage, records retrieved from the three databases were assessed against four inclusion criteria: (i) the article mentioned at least one of the search terms in the full text; (ii) the article was published in English; (iii) the full text was available; and (iv) the article was peer-reviewed. Records from EBSCOHost and ProQuest were managed directly, while records retrieved from Google Scholar were exported to EndNote for organisation and de-duplication. After consolidation across the three sources, 104 duplicate records were identified and removed.

At the eligibility stage, the 354 remaining records were assessed in full text for substantive topical focus. Keyword presence was treated as a necessary but not sufficient condition. Records were excluded when digital or online platforms were not the primary focus of the article during research ethics review processes. This accounted for most full-text exclusions (*n* = 347), with most excluded articles addressing research ethics more broadly, or digital tools used in research administration outside the ethics review context. Screening was conducted by the principal author, with the co-author providing supervisory verification of inclusion decisions and resolving uncertain cases through discussion.

A formal quality appraisal using a named instrument (such as CASP, MMAT, or JBI) was not undertaken. The seven included studies are methodologically heterogeneous, comprising empirical evaluations, a survey study, a workflow system description, a conceptual proposal on distributed ledger technologies, and a national policy framework, and no single appraisal tool is suited to assessing this range of study types. Instead, the included studies were informally assessed for relevance, methodological clarity, and contribution to the thematic synthesis. The implications of this approach are addressed in the Limitations section.

The search yielded 458 records. Following screening for relevance and removal of duplicates, seven studies met the eligibility criteria for inclusion. Rather than presenting a procedural audit of screening steps, this mini review synthesises the thematic contributions of the included studies, focusing on reported benefits, challenges, and implementation recommendations, as well as broader implications for institutional readiness and digital autonomy in ethics oversight.

Table 1 below summarises the included studies, outlines their key characteristics and primary focus areas, and serves as the foundation for the subsequent thematic analysis.

**Table 1:** Summary of eligible articles.

Year	Title	Author (s)	Focus of study
1. 2015	The satisfaction and use of Research Ethics Board information systems in Canada (2015)	Detlor, B., & Wilson, M. J.	Surveys research ethics personnel on their use and satisfaction with REB web-based systems, identifying factors that influence implementation and success.
2. 2015	Supporting ethical web research: A new research ethics review (2015)	Bowser, A., & Tsai, J. Y.	Develops a research ethics framework and submission system within Microsoft Research, specifically designed for web-based research.
3. 2017	Enhancing the efficiency and quality of African research ethics review processes through an automated review platform (2017)	Mokgatla, B., Bahati, P., & IJsselmuiden, C.	Evaluates an automated ethics review platform following its rollout and assesses the technological needs of Research Ethics Committees (RECs) in Africa.
4. 2018	Ethics governance outside the box: Reimagining blockchain as a policy tool to facilitate single ethics review and data sharing for the ‘omics’ sciences (2018).	Rahimzadeh, V.	Examines the use of distributed ledger technologies to enable single institutional ethics review of multisite collaborative studies, alongside the ethical, legal, and social implications of such applications.
5. 2021	A workflow system for managing ethical clearance in research work (2021)	Mbabe, W., Ajayi, O., Bagula, A., Leenen, L., & Schoeman, N.	Automates the ethical clearance approval process by developing a workflow system that addresses the inefficiencies of traditional manual review methods.
6. 2021	Developing quality and efficiency of IRB review under HRPP at a leading hospital in central southern China: A descriptive analysis of the first three years (2021)	Wang, X., Hahne, J., Li, L., Khoshnood, K., Yang, G., Yuan, H., & Liu, X.	Describes the implementation of an online IRB review platform, analysing submitted protocols and review processes over a three-year period.
7. 2024	A transformative solution to build effective, transparent, and resilient “fit-for-purpose” national health research ethics systems (2024)	Rani, M., Chawla, N., Wadhwa, N., Mathur, R., Jinks, T., Das, P., & Rijal, S.	Proposes a centralised national digital ethics review platform to address fragmentation and lack of harmonisation, focusing on transparency, reduced administrative burden, multi-site research enablement, and strengthened national research governance.

## Thematic synthesis of the literature

The small but growing body of literature on DPER reveals a field primarily concerned with procedural reform rather than governance transformation. Across the seven included studies, DPER are discussed as tools for modernising ethics review workflows, alongside reported implementation challenges, uneven institutional readiness, and emerging recommendations for system design and deployment. However, the evidence also highlights significant implementation challenges and uneven institutional and national readiness, as well as emerging recommendations for system design and deployment.

### Reported benefits of digital platforms for ethics review

The most consistent findings across the included studies (*n* = 6) are that DPER are associated with improvements in administrative efficiency and workflow management. Structured submissions, automated tracking, and centralised documentation contribute to reduced turnaround times, clearer audit trails, and improved communication among research stakeholders (4–9). Three studies further report that digitalisation reduces administrative errors through standardised data entry and automated protocol categorisation, with some noting reductions in operational costs where digital tools streamline repetitive tasks (4–6).

Beyond institutional-level gains, some authors extend the discussion to system-wide benefits. For example, Rani et al. (9) describe a proposed national digital ethics review platform that can harmonise review procedures across RECs, reduce duplication, facilitate multi-site research, and enable real-time tracking of performance metrics and research registries. Such models position digital platforms not only as workflow tools but also as infrastructure that enhances transparency, coordination, and resilience in national research governance. Similarly, Rahimzadeh (10) highlights how digital infrastructures can support single ethics review models and enhance coordination, transparency, and accountability across institutions.

Empirical evidence from African RECs further demonstrates that automated platforms improve standardisation, communication, and the management of multi-centre studies (5).

Collectively, these findings position DPER as a tool for addressing longstanding procedural inefficiencies in ethics review processes, while also signalling their potential role in broader system integration.

### Implementation and operational challenges

Despite these reported benefits, the literature highlights important challenges that complicate the adoption and sustainability of DPER. Usability concerns are frequently noted (*n* = 4), including difficulties with system interfaces, data entry accuracy, and adaptability to institutional workflows (4–6, 8). Prototype systems were associated with design limitations and user experience barriers that may undermine platform effectiveness if left unaddressed (4, 8).

Cost and implementation demand present additional constraints. Some studies (*n* = 4) emphasise that existing commercial platforms may be expensive or time-consuming to deploy, prompting institutions to consider custom-built systems instead (4–6, 8). However, developing and maintaining tailored systems requires technical expertise and ongoing institutional support, which may not be uniformly available. At a broader level, system-wide proposals further highlight the resource-intensive nature of implementing and scaling DPERs, including the need for coordinated infrastructure and capacity building (9).

Infrastructure disparities further shape adoption. In several African contexts, research ethics committees operate within environments characterised by uneven connectivity, limited technical capacity, and resource constraints (5). These conditions raise concerns about equitable access to digital systems and the risk that digitisation may amplify institutional inequalities if readiness varies significantly across settings.

### Practice and system development recommendations

The literature emphasises the importance of aligning DPER with institutional context and stakeholder needs. Studies (*n* = 3) suggest that system design should involve research stakeholders, including committee members and administrative staff, to ensure usability and acceptance (5, 6, 8). Customisation and adaptability are highlighted as key factors in successful implementation, with a recognition that a single standardised system may not suit all institutional environments (5).

Rani et al. (9) propose moving beyond institution-specific tools toward centralised digital infrastructures that integrate submission and review workflows, registries, and performance monitoring into a single national platform. Such approaches are framed as mechanisms for harmonising procedures, strengthening oversight resilience, and reducing fragmentation in research ethics governance. However, these proposals also introduce policy, coordination, and implementation challenges that extend beyond technical system design.

Recommendations also include strengthening data governance mechanisms, user training, and technical support structures to enhance sustainability (10). Further, Rahimzadeh (10) proposes innovative technological approaches, such as distributed ledger technologies, to improve transparency and traceability in review processes, although such proposals introduce additional legal and policy considerations.

Overall, recommendations tend to focus on improving system performance and implementation processes rather than fundamentally rethinking the governance architecture of ethics oversight.

## Discussion

The evidence reviewed suggests a clear imbalance in the conceptualisation of digital platforms for ethics review (DPER) in the literature. This limited number of studies represents a key finding, rather than an incidental outcome of the search. It indicates that peer-reviewed research on DPER as a defined empirical phenomenon remains in an early, underdeveloped stage. The eligibility threshold was deliberately set to include only studies that engaged directly with digital platforms for ethics review rather than studies on digital governance, e-governance, e-government or platform regulation more broadly. While these adjacent studies offer conceptual resources for theorising digital ethics oversight, they do not constitute empirical evidence on DPER and were therefore excluded from the synthesis to preserve analytical specificity. This scope shapes what the review can and cannot claim. With only seven eligible studies published over more than a decade across very different institutional settings, this review is best understood as an early survey of an emerging field rather than a synthesis of established evidence. The findings describe how DPER are currently being studied largely as workflow tools, with limited attention to governance rather than offering generalisable conclusions about whether DPER work, or whether RECs are ready for digital autonomy. Evidently, the studies selected are informative. It shows that more empirical studies, clearer shared terminology, and broader methodological engagement are needed before stronger claims can be made.

Although DPER is located within a broader ‘digital governance turn’ which is affecting institutions globally, the literature reviewed here treats them in relative isolation from these wider debates. Most studies frame digitalisation as a response to procedural inefficiencies and position digital systems primarily as workflow solutions (4, 6, 7). Far less attention is given to the broader governance conditions required for RECs to function effectively within DPERs.

These findings reveal a disconnect between digital adoption and digital autonomy. While several studies document successful deployment of digital platforms and associated efficiency benefits (4, 5), these outcomes do not necessarily indicate institutional readiness for sustained digital operation. Factors such as infrastructure stability, institutional support structures, stakeholder involvement, and data protection mechanisms are discussed unevenly and often treated as implementation considerations rather than as foundational elements of ethics review governance.

The literature also reflects variability in the scale at which digital solutions are imagined. Many studies focus on institution-specific systems to improve local review processes (4, 6). In contrast, emerging proposals for centralised national platforms reframe digital systems as components of research governance infrastructure, intended to harmonise procedures, integrate registries, and enable system-wide transparency and resilience (9). This shift in perspective highlights that digitalisation may alter not only workflows but also the organisational architecture of ethics oversight.

Despite these developments, key governance questions remain underexplored. Few studies examine how digital platforms influence decision-making authority, committee deliberation, or accountability pathways within RECs. Similarly, while digital tracking enhances traceability (10), its implications for the quality and integrity of ethical review are rarely assessed. As a result, digital transformation is often evaluated in terms of speed and organisation rather than the robustness of oversight.

Altogether, these themes suggest that the literature treats digitalisation primarily as a procedural modernisation, whereas the conditions associated with digital autonomy point to a deeper structural transition. Without explicit attention to governance readiness, digital platforms for ethics review risk optimising processes without strengthening the institutional foundations of ethical oversight.

Furthermore, while the evidence reviewed draws on studies from a range of institutional and national settings, several of the empirical studies on DPER implementation come from African contexts (2, 4, 5). This reflects both the inclusion of these studies in the eligible research and the analytical lens of this review, which engages with research ethics governance from a Global South perspective. Observations regarding administrative efficiency, communication, and documentation recur throughout the review and are broadly applicable to RECs operating in diverse settings. Findings relating to infrastructure constraints, uneven digital capacity, and the risks of amplifying institutional inequality are most clearly evidenced in African and other resource-constrained contexts, though they are likely relevant wherever RECs operate with limited technical or institutional support. Indeed, attending to contexts where institutional capacity is uneven sharpens, rather than narrows, the analysis by foregrounding conditions that are easily overlooked in literatures dominated by well-resourced settings.

## Limitations of the mini review

The findings of this review should be interpreted considering its limitations. First, the search strategy was confined to a defined set of electronic databases. As a result, relevant studies, particularly those addressing digital autonomy in ethics review under different terminology, may not have been captured.

A second limitation relates to the search terms used. Although care was taken to select keywords closely aligned with the concept of ‘digital platforms for ethics review (DPER),’ there is currently no standardised terminology in research ethics scholarship. This may have constrained the retrieval of studies that use alternative languages to describe similar systems. Nevertheless, the breadth of articles identified suggests that the search strategy was sufficiently sensitive across interdisciplinary domains. The absence of a common term also highlights a field-level need for clearer and more consistent terminology to describe digital platforms in ethics review processes.

The review was further limited to English-language publications, which may have skewed representation toward contexts where English dominates scholarly communication, potentially underrepresenting experiences from other regions.

The empirical evidence base is also weighted toward certain contexts, particularly African RECs and a small number of high-income institutional settings and does not capture the full range of regional and institutional experiences with DPER. Findings should therefore be interpreted as indicative of the orientations of existing research rather than as comprehensive across all contexts.

Overall, this review sought to map the current state of knowledge on digital autonomy in research ethics review. It aimed to characterise the scope and nature of existing scholarship, identify conceptual and empirical gaps, and inform future research directions in the digital transformation of ethics oversight.

## Conclusion

This mini review examined the emerging literature on digital platforms for ethics review and assessed the current evidence on readiness for digital autonomy in research ethics oversight. While digital systems are consistently associated with improvements in workflow efficiency, communication, and documentation management, the evidence base remains limited and largely focused on operational performance.

A key insight is the distinction between digital adoption and digital autonomy. The use of digital tools does not automatically mean that research ethics committees are prepared to function effectively within digital environments. Readiness depends on broader institutional and systemic conditions, including infrastructure capacity, stakeholder involvement, institutional support, and adequate privacy and security safeguards. These factors indicate that digital autonomy represents a governance state rather than simply the presence of digital platforms.

Emerging models that frame digital systems as components of research governance infrastructure suggest a shift toward more integrated and coordinated oversight approaches. However, such developments also introduce policy, implementation, and capacity challenges that remain insufficiently examined.

Overall, digital platforms for ethics review hold clear potential to strengthen research ethics review, but current scholarship provides limited evidence that systems are fully prepared for digital autonomy. Future work should therefore move beyond efficiency-focused evaluations to examine how digital transformation interacts with institutional readiness, governance structures, and the deliberative dimensions of ethical oversight.
